# Straw Wine Melanoidins as Potential Multifunctional Agents: Insight into Antioxidant, Antibacterial, and Angiotensin-I-Converting Enzyme Inhibition Effects

**DOI:** 10.3390/biomedicines6030083

**Published:** 2018-08-02

**Authors:** Vlasios Goulas, Demetra Nicolaou, George Botsaris, Alexandra Barbouti

**Affiliations:** 1 Department of Agricultural Sciences, Biotechnology and Food Science, Cyprus University of Technology, 3036 Lemesos, Cyprus; demetra2604@gmail.com (D.N.); george.botsaris@cut.ac.cy (G.B.); 2 Department of Anatomy-Histology-Embryology, Faculty of Medicine, University of Ioannina, 45500 Ioannina, Greece; abarbout@cc.uoi.gr

**Keywords:** angiotensin I converting activity, Commandaria wine, *Escherichia coli*, *Listeria*, radical scavenging activity, *Salmonella*

## Abstract

Numerous studies provide robust evidence for a protective effect of red wine against many diseases. This bioactivity has been mainly associated with phenolic fractions of wines. However, the health effects of melanoidins in red sweet wines has been ignored. The goal of the present work was to unravel the antioxidant, antimicrobial, and angiotensin-I-converting enzyme (ACE) inhibitory properties of straw sweet wine melanoidins. Results demonstrated that melanoidins have a potential antioxidant activity, determined by 2,2-Diphenyl-1-picrylhydrazyl (DPPH) and Ferric reducing antioxidant power (FRAP) assays. The antimicrobial activity of melanoidins was also tested against *Listeria monocytogenes*, *Salmonella* Enteritidis, and *Escherichia coli*. Minimum inhibitory concentration (MIC) of isolated melanoidins against three bacterial strains ranged from 5 mg mL^−1^ to 10 mg mL^−1^. Finally, the ACE inhibitory effect of isolated melanoidins was evaluated, as it is linked with antihypertensive activity. Results showed that they have ACE-inhibitory activity ranging from 58.2 ± 5.4% to 75.3 ± 6.4% at a concentration level of 2 mg mL^−1^. Furthermore, the chemical properties of isolated melanoidins were determined. Results demonstrated that the skeleton of straw wine melanoidins is mainly composed of carbohydrates, and bear significant numbers of phenolic compounds that may play critical roles in their functional properties. Overall, this study describing the chemical composition and functional properties of melanoidin fractions isolated from a straw wine highlights that they can be exploited as functional agents for multiple purposes. Finally, melanoidins are an unexplored source of bioactive molecules in straw wines except from polyphenols that contribute to the health effects.

## 1. Introduction

Melanoidins are polymeric and brown-colored macromolecules originated by the interaction between carbohydrates (mainly reducing sugars) and compounds with free amino groups, namely amino acids, and proteins. The latter non-enzymatic transformation is well-known as Maillard reaction [1]. These compounds are considered high-molecular weight polymers, although their structures are not fully elucidated yet. In general, melanoidins are hydrophilic nitrogen compounds with anionic charge, and their molecular weight ranges from few thousands of Daltons to more than 100 kDa [2,3]. Furthermore, the phenolic compounds are associated to the surface of isolated melanoidins, as previous studies have reported for melanoidins that are isolated from coffee [4], cocoa [5], beer [6], and sweet wine [7]. They are usually attached to the structure of melanoidins via non-covalent bonds contributing to their functional and biological properties.

Melanoidins as a result of Maillard reaction have been a major challenge in the food industry, since they contribute to the flavor, color, and texture of foods. Although, the first perception considered melanoidins as an inert or anti-nutritional constituent, a significant number of health-promoting properties of melanoidins have been documented. The antimicrobial [8,9], antioxidant [10,11], antihypertensive [12], and prebiotic activities [13,14] of melanoidins are the most promising among them. Recently, a significant contribution of melanoidins to the daily antioxidant capacity intake was also revealed. In particular, the contribution can reach up to 20%, which is a large amount compared to the 10% belonging to the joint daily intake of well-known vitamins C and E [15].

Sweet wines are considered as a source of melanoidins since they are formed during either dehydration process of grapes or heat concentration of grape musts. The presence of melanoidins has been confirmed in Italian “vino cotto” wine [16] and Spanish sweet wines [17]; these sweet wines are produced by boiling the grape musts to concentrate sugar content. Previous studies have been mainly focused on the antioxidant properties of sweet wines melanoidins; they exert a potent antioxidant activity with 4160 µmol Trolox per serving [15].

Commandaria is an amber-colored straw wine produced on the island of Cyprus using sun-dried grapes. Commandaria is exclusively produced through the fermenting process of must obtained from sun-dried grapes of two indigenous grape cultivars, namely “Xynisteri” (white grapes) and “Mavro” (red grapes). In the traditional Commandaria production process, grapes are spread on nets placed in large open sites with suitable orientation and gentle slope for ca. two weeks, depending on the climatic conditions, in order to be dehydrated [18]. During the dehydration period, grapes under sun exposure are heated intensively reaching 50–55 °C, boosting the formation of melanoidins. The duration and temperature of sun exposure is expected to influence the melanoidin composition. Furthermore, the postharvest dehydration methods of grapes are also thought to influence the melanoidin content in Commandaria wines [19]. The aim of the present work was to study the melanoidin fraction of a straw sweet wine like Commandaria. Furthermore, possible health effects were also studied, as the antioxidant, antimicrobial, and antihypertensive potency of isolated melanoidins were also investigated.

## 2. Materials and Methods

### 2.1. Chemicals and Samples

Gallic acid, ascorbic acid, phenol, sodium carbonate, sodium acetate, iron (III) chloride, 2,2-Diphenyl-1-picrylhydrazyl (DPPH), 2,4,6-Tris(2-pyridyl)-s-triazine (TPTZ), hippruric acid, angiotensin I-converting enzyme (ACE, EC 3.4.15.1), hippuryl-his-leu hydrate (HHL) and common solvents and inorganic acids were purchased from Sigma Chemical Co. (St. Louis, MO, USA). Folin-Ciocalteu reagent, sodium chloride and di-Sodium tetraborate decahydrate were obtained from Merck (Darmstadt, Germany); while brain heart infusion (BHI) broth was purchased from Oxoid Australia (West Heidelberg, VIC, Australia).

Two bottles of four aged and four non-aged Commandaria wines were kindly donated by local producers and were used to isolate melanoidins.

### 2.2. Isolation of Melanoidin Fractions

The isolation of melanoidins from Commandaria wines were performed according to previous studies about Spanish sweet wines [17]. Approximately, 10 mL of wine was transferred into dialysis tubing which retain molecules bigger than 12,000 Da, and it was placed in a glass vessel with 1 L of deionized water at 4 °C. The solution was stirred for 12 h. This procedure was repeated twice. The volume of wine which remained in the dialysis tube was freeze-dried and the dry matter was stored at −20 °C until needed.

### 2.3. Spectroscopic Analysis

Melanoidin fractions were diluted with deionized water 1/5 (*v*/*v*) and their absorbance was measured at 345 nm, 420 nm, and 440 nm [7].

### 2.4. Determination of Total Phenolic Groups Content

The total phenolic groups content of melanoidins was determined using the Folin–Ciocalteu method. Briefly, 790 μL of deionized water, 10 μL of each melanoidin fraction (2.5 mg mL^−1^), and 50 μL of Folin–Ciocalteu reagent were mixed. After 1 min, 150 μL of 20% *w*/*v* NaCO_3_ was added, and the mixture was mixed and allowed to stand at room temperature. After 120 min, the mixture was monitored at 760 nm and the phenolic content was calculated as mg gallic acid equivalents (GAE) g^−1^ of melanoidin [10].

### 2.5. Determination of Total Sugar Content

The carbohydrate content of melanoidins was quantified using the phenol-sulfuric acid method after mild acid hydrolysis [10]. Melanoidin fractions at a concentration level of 2.5 mg mL^−1^ were mixed with 1 N HCl for a period of 2 h at 105 °C. Afterwards, the mixture was cooled at room temperature and diluted 10 times with deionized water and filtered. In a next step, an aliquot of mixture (100 μL) was react with 100 μL of 5% *w*/*v* phenol. Then, 1 mL of 96% *w*/*v* H_2_SO_4_ was added resulting in a colorful product, and the absorbance was read at 500 nm. Results were expressed as mg glucose equivalents g^−1^ melanoidin.

### 2.6. Determination of DPPH Radical Scavenging Activity

An 80 µL aliquot of melanoidin fraction (2.5 mg·mL^−1^) sample was mixed with 1 mL of DPPH 74 mg·L^−1^ in methanol. The mixture was shaken and incubated for 1 h in the dark. Then, the absorbance was measured at 520 nm. The radical scavenging activity was calculated using a standard curve of ascorbic acid and expressed as μmol ascorbic acid (AsA) equivalents g^−1^ melanoidin [8].

### 2.7. Determination of Ferric Reducing Antioxidant Power (FRAP)

An aliquot of 150 μL of melanoidin fraction (2.5 mg·mL^−1^) was mixed with 3 mL of FRAP solution and incubated at 37 °C for 30 min. The FRAP solution contained 2.5 mL 0.3 mol L^−1^ acetate buffer (pH 3.6), 250 μL 10 mmol·L^−1^ TPTZ, and 250 μL 40 mmol·L^−1^ FeCl_3_·6H_2_O. After reacting, the absorbance was measured at 593 nm. The antioxidant activity was expressed as μmol FeSO_4_ g^−1^ melanoidin [18].

### 2.8. Determination of Antimicrobial Potency

The influence of different concentrations (5–30 mg·mL^−1^) of melanoidins on the growth of *Listeria monocytogenes* NCTC 7973*, Salmonella enterica subsp. enterica serovar* Enteritidis NCTC 5188, and *Escherichia coli* ATCC 11775 was determined by the microtiter plate method [20]. Overnight suspensions of the three bacterial strains were growth at 37 °C in BHI broth until a concentration of 10^8^ colony forming units (cfu) mL^−1^ was reached. Then, 250 µL of bacterial cell suspensions were inoculated into a sterile 96-well microplate. Subsequently, 50 µL of melanoidins at different concentrations were added. Melanoidins were re-suspended in sterile distilled water and filtered using a Millipore sterile 0.22 μm filter unit prior to use. Microbial growth kinetic was recorded on a Multiskan™ GO Microplate Photometer (Thermo Fisher Scientific, Vantaa, Finland). The mixture was shaken for 1 min at 150 rpm and incubated at 37 ± 1 °C for 24 h. The turbidity of mixtures was measured as having an absorbance at 600 nm, and were read every 10 min. An agitation for 10 s was performed to achieve homogeneous suspensions before each measurement. All experiments were performed in triplicate. The minimum inhibitory concentration (MIC) was defined as the lowest concentration of melanoidins at which bacterial growth was inhibited compared to the control.

### 2.9. Determination of Angiotensin-I Converting Enzyme (ACE) Inhibitory Activity

An aliquot of 100 μL of melanoidin fraction (2 mg·mL^−1^) was mixed with 80 μL of ACE solution (0.1 U·mL^−1^) containing 150 mM borate buffer (pH = 8.3) with 300 mM sodium chloride and allowed at 37 °C for 10 min. Afterwards, 100 μL of 5.0 mM HHL solution was added to the mixture. The reaction was incubated at 37 °C for 60 min and then it was stopped with 250 μL of 1 M HCl. The hippuric acid formed was detected and quantified by high pressure liquid chromatography (HPLC) method. A Waters series HPLC 1525, equipped with vacuum degasser, binary pump, autosampler, thermostated column compartment, and dual wavelength absorbance detector (Waters Corporation, Milford, Ireland) was used. After filtration with syringe filters (0.45 μm), 20 μL of mixture was injected on a Spherisorb^®^ ODS2 (Waters Corporation, Milford, Ireland) column (250 mm, 4.6 mm i.d., 5 mm). The solvents used were 10 mM phosphoric acid in water (solvent A) and methanol (solvent B) with the following linear gradient 0 min 100% A, 0–8 min 40% A, 8–13 min 0% A, and 13–18 min 100% A. The flow rate was fixed at 1 mL·min^−1^ and the absorbance was recorded 228 nm. Results were expressed as the percentage inhibition of enzyme activity [21].

### 2.10. Statistical Analysis

Statistical analysis was performed using the software package SPSS v20.0 (SPSS Inc., Chicago, IL, USA) and the comparison of averages of each treatment was based on the analysis of variance (one-way ANOVA) according to Tukey’s test at significance level 5% (*p* ≤ 0.05).

## 3. Results and Discussion

### 3.1. Chemical Characterization of Commandaria Melanoidins

Spectroscopic parameters of isolated melanoidins are listed in Table 1. Previous studies on wine melanoidins reported that the absorbance at 345 nm could be used as a measure of melanoidin content, while the absorbance values at 420 nm and 440 nm are used as browning index, which is strongly affected by wine melanoidin content [8,17]. The values of absorbance at 345 nm ranged between 0.180 and 0.525, at 420 nm and 440 nm from 0.066 to 0.234 and from 0.061 to 0.208, respectively, highlighting a great diversity in melanoidin contents in Commandaria wines. This diversity can be attributed to (i) the inconstant dehydration conditions in open nets, (ii) the different winemaking techniques, and (iii) the production of Commandaria from white and/or black grapes. Results also showed that the aging of Commandaria wines plays an important role in the formation of melanoidins. In particular, the mean values of absorbance at 345 nm of melanoidins from aged wines was 0.441 ± 0.091; whereas the corresponding mean values of melanoidins from non-aged wines was 0.238 ± 0.054. Browning indexes (A_420_, A_440_) also confirmed a higher melanoidin content in aged wines than non-aged ones. A similar effect of oak aging on the formation of melanoidins was described for traditional balsamic vinegar [10].

Table 1 also shows the concentration of sugars that were released after mild acidic hydrolysis of Commandaria melanoidins. The total sugar contents were from 452 mg glucose equivalents g^−1^ melanoidins to 612 mg glucose equivalents g ^−1^ melanoidins, suggesting that their structures are like melanosaccharides [15]. This structure is expected as the concentration of reducing sugars (glucose and fructose) is higher than 20% *w*/*w* in grape must at harvest, while their concentrations reach up to 36% *w*/*w* in grape must after sun-drying [18]. Results also demonstrated that wine aging had no effect on the sugar content of melanoidins; they compose the skeleton of melanoidins which is mostly framed during dehydration of grapes.

A great variety of phenolic content in Commandaria melanoidins was also found. Total phenolic content varied between 64.7 ± 3.3 mg and 165.2 ± 14.7 mg GAE g^−1^ melanoidin. The production of Commandaria wines from white and black grapes may influence this range of values; it is well known that black grapes contain higher amounts of phenolic compounds than white grapes [22]. The phenolic groups content in melanoidins is similar to Spanish sweet wines melanoidins (>100 mg GAE g^−1^ melanoidin), although the latter are produced via boiling process of the grape must. The high phenolic content also confirms that isolated melanoidins look like melanosaccharides as they have higher amounts of phenolics in comparison to melanoproteins [15]. Surprisingly, the mean value (84.3 mg GAE g^−1^ melanoidin) of phenolic groups content in melanoidins from aged wines is lower than the mean value (114.7 mg GAE g^−1^ melanoidin) of phenolics in melanoidins originating from non-aged wines. The participation of phenolic units in oxidation reaction during aging may explain the aforementioned depletion of phenolic content in melanoidins. Moreover, the loss of some monomer and oligomeric phenolic compounds during wine aging has been described [23]. Furthermore, this difference in phenolic groups content is not significant statistically. Regarding the nature of attached phenolic compounds, previous studies demonstrated that the most abundant phenolics are usually attached to the surface of melanoidins. In particular, chlorogenic acid and catechins are found in coffee and chocolate melanoidins, respectively [2]. Similarly, the must obtained from sun-dried grapes of two indigenous cultivars destined for the production of Commandaria wine are rich in the derivatives of hydroxybenzoic and hydrocinnamic acids like protocatechuic acid glucoside, vanillyl alcohol glucoside, caftaric acid, gallic acid hexoside, etc. [18].

### 3.2. Antioxidant Activity of Commandaria Melanoidins

**Figure 1:**
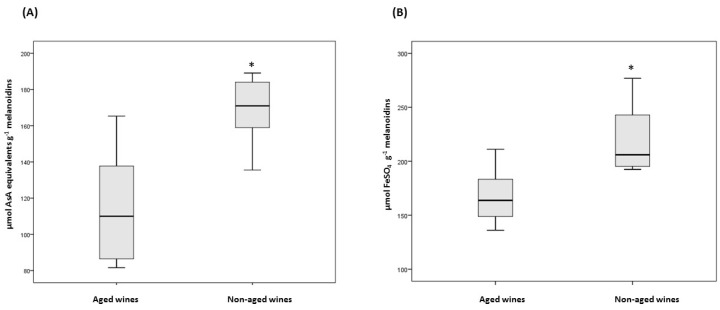
Antioxidant potency of melanoidins isolated from aged and non-aged Commandaria wines evaluated with (**A**) 2,2-diphenyl-2-picrylhydrazyl (DPPH) assay and (**B**) ferric reducing antioxidant power (FRAP) assay. Significant differences (*p* < 0.05) for aged and non-aged Commandaria wines are indicated with *.

The antioxidant properties of Commandaria melanoidins were tested using two in vitro assays, namely DPPH and FRAP assays, as a recent study mentioned that melanoidins contribute to 20% of the total antioxidant capacity intake in the Spanish population [15]. A multiple assays approach was preferred to assess the antioxidant activity of melanoidins, since a single assay can give only a controversial suggestion of this and must be interpreted with some caution [24]. In the present study, the antioxidant assays used are based on different mechanisms that measure the antioxidative effect of melanoidins. The DPPH assay is based on the ability of antioxidants to act as radical scavengers and the FRAP assay measures the ability of antioxidants to perform as reducing agents [25]. The antioxidant activity of Commandaria potency ranged between 90.7 to 185.7 μmol AsA equivalents g^−1^ melanoidins for the DPPH assay and 148.6 to 260.6 μmol FeSO_4_ g^−1^ melanoidins for the FRAP assay (Figure 1). Both assays demonstrate that melanoidins isolated from non-aged Commandaria wines present higher antioxidant potency than melanoidins originated from aged wines. A close correlation (*r* = 0.785) between DPPH and FRAP values verifies this finding. The median DPPH values for aged and non-aged Commandaria wine melanoidins were 113.2 μmol AsA equivalents g^−1^ melanoidin and 171.0 μmol AsA equivalents g^−1^ melanoidin, respectively; whereas the corresponding values for the FRAP assay were 164.2 μmol FeSO_4_ g^−1^ melanoidin and 226.0 μmol FeSO_4_ g^−1^ melanoidin. In an attempt to correlate antioxidant activity with chemical properties of Commandaria melanoidins, the correlation coefficients were calculated. Results showed that the phenolic compounds mainly contributed to the antioxidant activity of melanoidins (*r* = 0.899 for FRAP assay, *r*= 0.591 for DPPH assay). In addition, spectroscopic parameters cannot be used for indicators of antioxidant activity. Finally, the aging of Commandaria wines negatively influences the antioxidant properties of isolated melanoidins. The latter is linked with the depletion of phenolic groups in melanoidins after aging of wines. Overall, the straw wine melanoidins can be considered as an alternative antioxidant source for human nutrition, although the contribution of wine to the daily average intake of melanoidins is low. In general, bakery and coffee products are the main sources of melanoidins due to their consumption on a daily basis [3]. However, these findings show that sun-dried grapes (raisins) may contain significant amounts of antioxidant melanoidins.

### 3.3. Antimicrobial Potency of Commandaria Melanoidins

**Figure 2:**
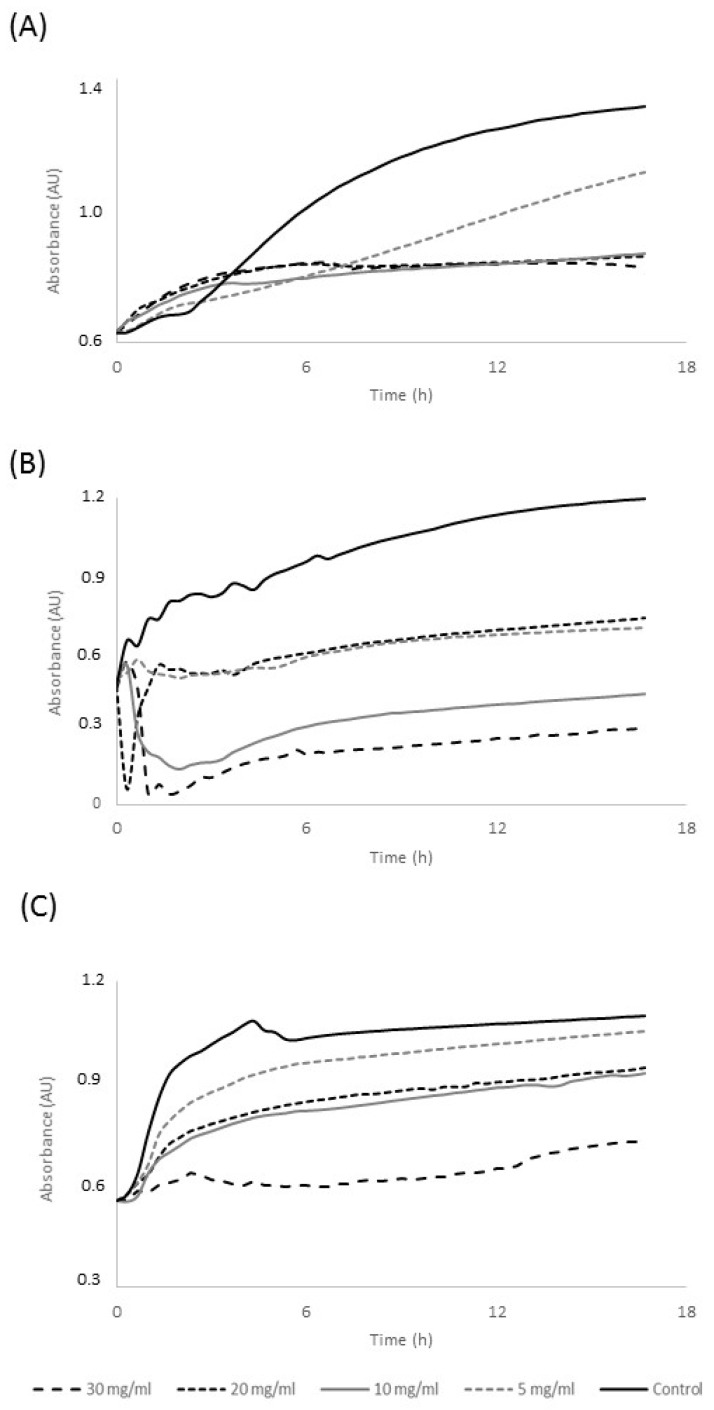
Typical dose-dependent inhibition profile of Commandaria melanoidins over (**A**) *Listeria monocytogenes*, (**B**) *Salmonella* Enteritis, and (**C**) *Escherichia coli*.

The antimicrobial activity of Commandaria melanoidins was measured against three different bacterial strains of significant importance to the food industry. Figure 2 presents a classical dose-dependent inhibition profile of Commandaria melanoidins over *Listeria monocytogenes*, *Salmonella enterica* subspecies *enterica* Enterititis, and *Escherichia coli*. A decrease of the turbidity (A = 600 nm) was monitored as the concentration of melanoidin fractions was increased, indicating inhibition of bacterial growth. The concentration defined as the MIC for each bacterial strain was determined. Table 2 summarizes the MIC values of Commandaria melanoidins against the three bacterial strains tested; MIC values ranged from 5 mg mL^−1^ to 10 mg mL^−1^. Similar findings have been previously reported for melanoidins and isolated from coffee, beer, and sweet wine [9,20].

Although significant differences in the spectroscopic and chemical properties of melanoidins were found, they did not affect the antimicrobial potency of Commandaria melanoidins. The spectroscopic indexes and total phenolic group contents of melanoidins isolated from aged and non-aged wines vary significantly, but these chemical properties did rather not influence their antimicrobial potency. The assessment of inhibitory effect of melanoidins at concentrations lower than 5 mg·mL^−1^ could pinpoint differences in their antimicrobial potency between melanoidins studied. However, previous works reported MIC values for food-isolated melanoidins ranging between 5 mg·mL^−1^ and 50 mg·mL^−1^ for *E. coli* strains as well, and MIC value against *Salmonella* Typhimurium at 4.5 mg·mL^−1^ [9,20]. Regarding their bioactivity, the antimicrobial effects of Commandaria melanoidins are comparable with natural polymers such as chitosan [26], lactofferin [27], nisin, and polymixin [28]. The antimicrobial activity of melanoidins is rather correlated with their metal-chelating properties. In particular, three mechanisms have been proposed to explain the antimicrobial activity of high molecular weight melanoidins. At low concentrations, melanoidins act as a bacteriostatic agent by binding iron from the culture medium. More specifically, the bacterial strains that are able to produce siderophores for iron acquisition may be susceptible to melanoidins, which can chelate the siderophore-Fe^3+^ complex. Melanoidins can also exert bactericide activity at high concentrations as they can bind Mg^2+^ cations from the outer membrane, boosting the disruption of the cell membrane and allowing the release of intracellular molecules [9].

### 3.4. ACE Inhibitory Effect of Commandaria Melanoidins

ACE is among the principal components of the renin–angiotensin system. It is found in two different isoforms possessing two and one zinc-containing active sites, respectively. ACE catalyzes the hydrolysis of angiotensin-I peptide and plays a crucial role in the release of angiotensin-II vasopressor peptide in blood [29]. Inhibitors of ACE are considered as candidate agents for treatment of many diseases such as hypertension, heart disease, diabetic neuropathy, and atherosclerosis [30].

**Figure 3:**
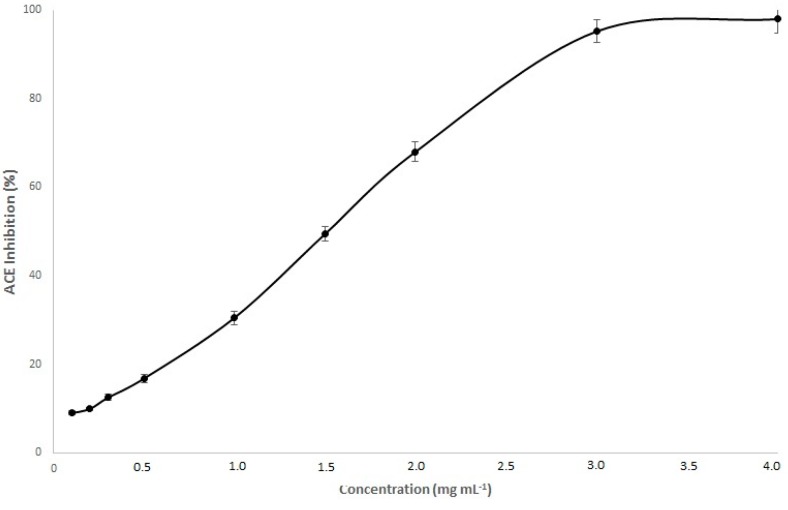
Dose response inhibition of angiotensin-I-converting enzyme inhibitory activity by Commandaria melanoidins.

At first, a dose-response inhibition of ACE activity of a melanoidin isolated from non-aged Commandaria wine was carried out in order to check the most appropriate concentration of melanoidin (Figure 3). A sigmoidal curve was found as previous work described for melanoidins and commercial antihypertensive drugs [12]. The concentration of melanoidins at 2 mg·mL^−1^ was selected since (i) it points out in a linear range and (ii)as allows the comparison with isolated melanoidins from other sources [8,12].

ACE inhibition percentages of Commandaria melanoidins are shown in Table 3. ACE-inhibitory activity values ranged from 54.4% to 79.8% for melanoidins at a concentration of 2 mg·mL^−1^. Melanoidins from non-aged Commandaria wines showed higher ACE inhibitory activity than melanoidins from aged ones. The latter is maybe correlated to phenolic group contents of melanoidins since phenolic compounds act as chelating agents [31]. The inhibitory activity of melanoidins can be partly linked with their metal chelating properties as ACE is a Zn-dependent enzyme [32]. There is strong evidence that melanoidins can act as metal chelating agents as their anionic nature permits them to chelate transition metals [33]. A previous study reported that ACE-inhibitory activity of melanoidins isolated from coffee, beers, and sweet wines ranged from 36.8% to 60.1% at the same concentration of 2 mg·mL^−1^ [12]; whereas the ACE inhibition percentages of melanoidins isolated from several amino acid–glucose model systems were between 28.7% and 64.3% [12]. Overall, the present study demonstrates the potential of isolated melanoidins to be exploited as antihypertensive agents. A new approach in hypertensive drug design is the use of cocktail formulations that contain more than one active component, acting synergistically, and therefore having optimized pharmacological benefits, thus melanoidins can be used as bioactive ingredients in these cocktail formulations [32].

The present work describes and characterizes the melanoidins originating from a dessert wine that is produced using sun-drying of grapes. Results showed a great diversity in chemical properties of isolated melanoidin fractions; the production of Commandaria wines using white and black grapes and the utilization of different enological techniques (e.g., alcohol fortification, duration of aging) may partly explain these findings. Regarding the bioactivity of melanoidin fractions, promising in vitro antioxidant, antimicrobial, and ACE inhibitory activities were found. The present study demonstrates that the unexplored melanoidin fraction in straw wines also contributes to the health effects of wine. Although the consumption of an alcoholic beverages in order to promote human health cannot be recommended, their utilization to formulate innovative functional foods stands as a challenging prospective for food industry. Finally, the present study shows that thermal processing of grapes and its products can be driven to the production of biologically active melanoidins.

## Figures and Tables

**Table 1:** Chemical properties (A_345_, A_420_, A_440_, sugar, and phenolic contents) of melanoidins isolated from aged and non-aged Commandaria wines. Significant differences (*p* < 0.05) for aged and non-aged Commandaria wines are denoted with *.

Wines	A_345_		A_420_		A_440_		Sugar Contents (mg GE g^−1^ m ^†^)		Phenolic Groups Contents (mg GAE g^−1^ m ^‡^)	
	Mean	Range	Mean	Range	Mean	Range	Mean	Range	Mean	Range
Aged	0.441 *	0.313–0.525	0.193 *	0.132–0.234	0.168 *	0.116–0.208	509.8	452.2–550.7	84.3	64.7–99.7
Non-aged	0.238 *	0.180–0.311	0.099 *	0.066–0.140	0.091 *	0.061–0.130	520.5	462.6–612.2	114.7	84.0–165.2
All	0.340	0.180–0.525	0.146	0.066–0.234	0.130	0.061–0.208	515.1	452.2–612.2	99.5	64.7–165.2

^†^ mg GE g^−1^ m: mg glucose equivalents g^−1^ melanoidin; ^‡^ mg GAE g^−1^ m: mg gallic acid equivalents g^−1^ of melanoidin.

**Table 2:** Minimum inhibitory concentration (MICs) of Commandaria wine melanoidins isolated from aged and non-aged Commandaria wines.

Microorganism	MIC (mg mL^−1^)	
	Aged Wines	Non-Aged Wines
*Listeria monocytogenes*	5	5
*Salmonella Enteritidis*	5	5
*Escherichia coli*	10	10

**Table 3:** Angiotensin I converting enzyme inhibitory activity of melanoidins at a concentration of 2 mg·mL^−1^ isolated from aged and non-aged Commandaria wines. Significant differences (*p* < 0.05) for aged and non-aged Commandaria wines were found.

Wines	Mean (%)	Range (%)
Aged	58.4	54.4–62.1
Non-aged	74.4	70.7–79.8
All	66.4	54.4–79.8
